# Seasonal, annual, and spatial variation in cereal prices in Sub-Saharan Africa

**DOI:** 10.1016/j.gfs.2020.100438

**Published:** 2020-09

**Authors:** Camila Bonilla Cedrez, Jordan Chamberlin, Robert J. Hijmans

**Affiliations:** a Department of Environmental Science and Policy, University of California, Davis, USA; b International Center for Tropical Agriculture (CIAT), Colombia; c International Maize and Wheat Improvement Center (CIMMYT), Nairobi, Kenya

**Keywords:** Food prices, Drought, Seasonality, Prediction

## Abstract

Local food prices are key indicators of food security and market conditions. Yet price data are often not available, particularly for rural areas of Sub-Saharan Africa. We compiled data from 168 markets to study spatial and temporal price variation. We found that prices slightly increase when the preceding growing season was dry. Across the continent, there is pronounced seasonal variation, with lowest prices 2–3 months after harvest and highest prices just before harvest. A predictive model explained 42% of the spatial variation in prices. Our results show that spatial and temporal price variation can be generalized and that prices can be estimated for unsampled locations or months. Such estimates may be used to improve the targeting of food security interventions and strengthen empirical policy-oriented research.

## Highlights

• We analyze food prices in 168 markets over 10 years across 30 countries in Africa.
• Seasonal price variation is pronounced, especially in southeast Africa.
• Generalizable spatial and temporal price variation enables predictive modeling.
• Predictive price mapping can enhance existing market information system data.
• Spatial price predictions should be helpful for understanding food security and targeting interventions.

## 1 Introduction

Households in developing countries spend a large share of their income on food, making them particularly sensitive to food prices and their fluctuations (Minot and Dewina, 2013). Moreover, the prices farmers receive for their marketed products is a determinant of the profitability of farming, thus affecting farm household welfare and incentives for investments. Data on market food price variation is thus important to understand the constraints and opportunities for interventions in food security and agricultural development. Yet available price data is typically sparse and may not adequately reflect spatial and temporal variation in the market prices faced by producers and consumers (Chamberlin and Jayne, 2013; Minten et al., 2013; Aggarwal et al., 2018).

There is a significant body of literature on temporal price variation (e.g. Manda, 2010; Aker, 2008; Gitonga et al., 2013; Kaminski et al., 2014; Hirvonen et al., 2015; Gilbert et al., 2017) and price formation in Sub-Saharan Africa (SSA) (Rashid and Minot, 2010; Brenton et al., 2014; Yami et al., 2017; Gitau and Meyer, 2018) and the role of economic remoteness in determining prices (e.g. Minten and Kyle, 1999; Stifel and Minten, 2008; Minten et al., 2013; Aggarwal et al., 2018). However, there has not been much empirical research on the broad spatio-temporal structure of market price variation with a view to predicting them at unobserved market locations and moments in time. An exception is Bonilla Cedrez et al. (2020) who modeled spatial variation in fertilizer prices in SSA to predict local fertilizer prices at locations for which no empirical data was available.

Because of the scarcity of local market price data (particularly for rural areas), researchers often assume that farmers across large areas face a common market price – sometimes defined as the average of regional or district prices available from household surveys, but often defined at the national level, even where such analyses are explicitly spatial in other dimensions (e.g. You et al., 2014; Kaminski et al., 2014). When sub-national prices are used, they are typically average prices for relatively large areas (e.g. Liverpool-Tasie et al., 2017) which may include significant internal heterogeneity, and they may not consider temporal (within or between year) price variation. Many studies are ambiguous about the nature of prices used in analysis. For example, Jama et al. (2017) describe using data from “market surveys” for each of the four countries in their study of fertilizer profitability, but do not specify the level of price observation or aggregation. Similarly, Magrini et al.’s (2017) analysis of household-level welfare responses to price changes uses nationally-representative survey data from several countries, but the authors do not specify the geographic level at which price indices are constructed for any of the countries in their analysis. Neither study addresses how the timing of the survey may have affected the prices observed, and the interpretation of the data.

In this paper, we analyze and model how local market prices for food in SSA vary between years (annual variation), within the year (seasonal variation), and locations (spatial variation) using time-series of market price data for cereals from 168 markets in 30 countries in SSA. The input data for our analysis are local market prices for staple food grains. As most crop production in SSA is under rainfed conditions, precipitation variability is an important source of annual price variation, also depending on the integration of local markets with regional and global markets (Aker, 2008; Raleigh et al., 2015). We therefore modeled annual price variation as a function of rainfall in the preceding growing season. Seasonal price variation can be strongly related to the cropping cycle, with high prices in the “lean-season” before harvest, and much lower prices just after harvest (Kaminski et al., 2014; Dillon, 2020; Burke et al., 2019), particularly in more remote areas (Ndiaye et al., 2015), in part because of higher transportation costs during the rainy season (Minten and Barrett, 2005). We modeled seasonal price variation across SSA as a function of time since harvest. In addition, we used spatial predictor variables (e.g. access to market and climate) to predict spatio-temporal price variation at unsampled locations.

The contribution of our analysis is twofold. First, we provide novel descriptions of the magnitude of the annual and seasonal market price variation in SSA. We show that prices vary considerably in time and within countries, underscoring the importance of time- and location-specific market price information. Our analysis indicates that using national average or capital market prices as proxies for local market prices is likely to diverge considerably from actual market prices in many areas. The second contribution of this paper is our description of relatively simple approaches to estimate prices at unobserved market locations (the expected market price for a given location if there were a market in that location). Spatio-temporal predictions of prices, produced by methods such as those we outline here, can support more accurate assessments of food security and the economic returns to farming, and thus help design more effective interventions and policies.

## 2 Materials and methods

### 2.1 Price data

We compiled sub-national monthly food price data for thirty countries in Sub-Saharan Africa from three data sources. We collected data for five countries (Chad, Malawi, Nigeria, Somalia, and Zimbabwe) for 2000 to 2018 from the FEWS-NET “Price Watch” system (FEWS-NET, 2019). The second data source was the FAO Global Information and Early Warning System (GIEWS, 2019) “Food Price Monitoring and Analysis” from which we obtained price data for 2000 to 2018 for twenty-one countries (Angola, Benin, Burkina Faso, Burundi, Chad, Central African Republic, Côte d’Ivoire, Djibouti, Ethiopia, Ghana, Guinea, Kenya, Madagascar, Malawi, Mali, Mozambique, Namibia, Niger, Nigeria, Rwanda, Sierra Leone, Senegal, Somalia, South Sudan, Sudan, Tanzania, Togo, Uganda, Zambia, Zimbabwe). The third data source was the Regional Agricultural Trade Intelligence Network (RATIN, 2019) from which we obtained data for five East African countries (Burundi, Kenya, Rwanda, Tanzania, and Uganda) for 2016. We only acquired one year of RATIN data, mainly to increase the number of markets (spatial coverage) for which we had data.

**Fig. 1:**
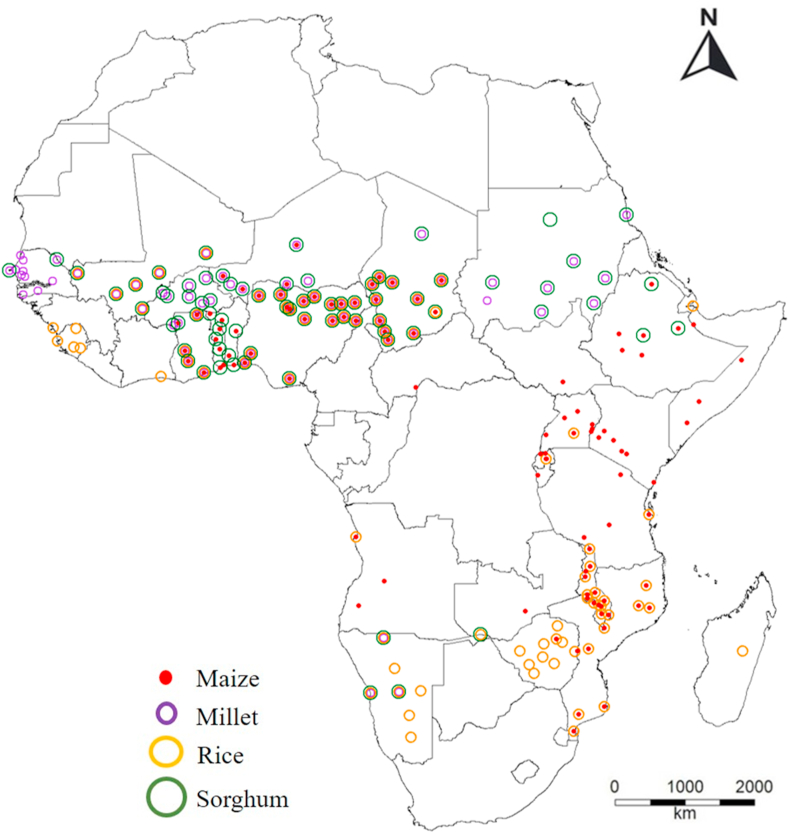
Locations of markets for which we obtained food price data for at least one of the following four products: maize, millet, rice, and sorghum.

All sources reported monthly price data for a particular market town. The foods reported varied by data source and country. We compiled monthly price data for maize, millet, sorghum, and rice because these are the major staple grains in SSA and the products for which the most observations were available (Fig. 1).

To allow for comparison of prices between years and countries, we converted prices from national currency to purchasing power and inflation adjusted United States dollars (USD) by dividing them by the monthly consumer price index (CPI) and monthly purchasing power parity (PPP) dollar value for each year according to the World Bank (World Bank, 2012). The CPI tracks the changes in the cost to the average consumer of acquiring a basket of goods and services that may be fixed or changed between years. Our base year was 2010 (CPI = 1). The PPP conversion factor, represent how much of a country's currency (expressed in USD using the international exchange rate) is required to buy the same amounts of goods and services in the domestic market as one USD would buy in the United States.

The final dataset consisted of 43,399 prices records, for 168 different market locations across the 30 countries. We had 13,278 observations for maize from 108 markets in 20 countries; 10,171 observations for millet from 74 markets in nine countries; 8989 observations for rice in 87 markets in 17 countries, and 10,961 observations for sorghum in 79 markets in 12 countries (Table 1S).

In 41 markets, both retail and wholesale prices were reported. In 89 markets, only retail prices were reported and in 40 markets, only wholesale prices were reported. We fit linear regressions models to study the relationship between reported retail and wholesale prices (USD kg^−1^) where both were available. We estimated that the wholesale price was 0.88 times the retail price for maize, 0.91 for millet, 0.94 for sorghum, and 0.83 for rice (slopes of regression lines). We adjusted the wholesale prices using these fractions to estimate retail prices for markets where only wholesale prices were available. For all other markets, we used the reported retail price data. After that, the total number of records was 39,481, containing 20,971 unique (market/year-month) price observations.

For markets where maize and other cereals were reported, we calculated the median price for each year and cereal and fit a linear regression model to study the relationship between maize prices and other cereals. Visual inspection showed good support for a linear relationship between the cereal prices (e.g. maize prices and other cereals). The Root Mean Square Error of the models varied between 0.22 and 0.25 USD kg^−1^ while mean crop prices varied between (0.74–1.72 USD kg^−1^) (see Appendix B for details). We used the fitted model to estimate a maize price equivalent for market where no maize price was available but where other cereals prices were reported, in order to expand the input data for spatial prediction of maize prices.

### 2.2 Timing of harvest

For each location we used a time series of monthly precipitation data (Harris et al., 2014) at a 0.5° (about 50 km) spatial resolution from 2000 to 2018. We determined the month of harvest (end of the growing season) for each market by using a simple rule: the harvesting starts in a month after a sequence of adjacent months that have at least a minimum amount of precipitation. This minimum amount of precipitation was location-specific and was defined as the observed median monthly precipitation for a year bounded between 30 and 90 mm (see Appendix C for the algorithm). In some regions, this rule creates single, and sometimes short, seasons. In other regions, there can be two growing seasons. We used a fixed cropping calendar to have a simple robust predictor and because, while the seasonal rainfall amount may vary considerably between years, the season duration in months changes little from year to year. The algorithm generated growing seasons that were very similar to published crop calendars (Sacks et al., 2010), but with much more spatial detail. For example, it showed two harvests seasons in areas where that is expected such as in northern Tanzania, Uganda, and Kenya. In some very dry regions (that is, with an annual precipitation lower than 200 mm) in Chad, Somalia, and Sudan the algorithm could not identify a growing season. These markets were removed from the seasonal price variation analysis.

### 2.3 Annual price variation

We studied the relationship between annual (between years) price variation and precipitation by fitting linear regression models where price was a function of the precipitation of the preceding growing season. In order to identify a dry year or a shock in prices related to the weather, we only considered the markets where we had at least 10 years of data, and a subset of 65 markets was used in this analysis. For each crop, market and “year” (from harvest to harvest, which is less than 12 months if there are multiple harvests per year), we computed the median price and the total precipitation. We detrended the price data by market and we selected the best out of three regression models of price as a function of year (intercept-only, first, and second-degree polynomial) using AIC. Finally, we fit a linear regression model between detrend price data and total precipitation in the preceding season and we analyzed the results by plotting the slope of these regression lines against average annual rainfall for each market.

### 2.4 Seasonal price variation

For each market, month and crop, we created a temporal price index computed as the twelve average monthly prices divided by the median observed price. Thus, for each month we obtained a value that represents how much the price in a month was lower or higher than the median price in each market. We fit linear regression models where price index was a function of month since harvest to study the relationship between seasonal price and crop cycle in relative terms. In regions with a single growing season, the maximum number of months since harvest is 11, and in regions with multiple growing seasons it is less; e.g. in Ethiopia, Uganda, and Kenya maximum number of months since harvest varied between 6 and 11 depending on the market.

### 2.5 Spatial price prediction

#### 2.5.1 Temporal price index

To study the spatial variation in the seasonal variation of food prices, we use the *temporal* price index described in the previous section. The index indicates at which time of the year prices are lower, equal, or higher than the annual median price for that location and allows us to compare temporal price variation in space while, controlling for price differences between locations. We fitted a Random Forest model to predict the temporal price index across Sub-Saharan Africa for each month. In the model, temporal price index was a function of longitude, latitude, precipitation, and month. The predictor variables were organized as raster data sets with a 5-arc minute (~9 km) spatial resolution. While our modeling framework is defined at this spatial resolution, our spatial summary of seasonal rainfall is calculated over an area defined by a 50 km buffer around each market location. We used the Random Forest algorithm as implemented in the *R*-package ‘randomForest’ (Liaw and Wiener, 2017). The spatial data was handled with the “raster” package (Hijmans, 2018). We computed Pearson's correlation coefficient and Root Mean Square Errors (RMSE) as test statistics to evaluate the model. We compared the RMSE with the RMSE of a Null-model (TE_0_) in which we assumed no temporal price variation by computing relative RMSE as TE_R=_1-(TE_I_/TE_0_). TE_R_ expresses how much better the interpolated predictions are relative to using a single national price over the months.

#### 2.5.2 Spatial price

We also modeled the actual spatial price variation, focusing on maize prices since maize is the most important staple grain in SSA. In this case, we compared maize prices as observed with estimated maize prices from the regression models described in Section 2.1. We used Random Forest to build a model of the maize price (USD kg^−1^) as a function of location (longitude and latitude), access to market and annual precipitation. Our market access indicators were the estimated travel time to the nearest town with 20,000 to 50,000 inhabitants, 200,000 to 250,000 inhabitants, and 1 to 5 million inhabitants (Nelson et al., 2019). Our measure of annual precipitation (mm) is from WorldClim v2 (Fick and Hijmans, 2017). We used the fitted model to predict maize prices across SSA, following a similar approach that was used to estimate fertilizer prices in SSA (Bonilla Cedrez et al., 2020).

### 2.6 Comparison between annual, seasonal, and spatial price variation

We computed annual, seasonal, and temporal price variation in countries with more than one market in order to compare the amounts of spatial, seasonal, and annual variation. For the annual variation, we computed the average national price by year and markets and then computed the price range (difference between the maximum and minimum price). For the seasonal variation, we computed the monthly average price in each market and then the seasonal price range, and averaged this by country. For the spatial variation, we computed the average price by market and then computed the price range for the country.

## 3 Results

### 3.1 Annual price variation

**Fig. 2:**
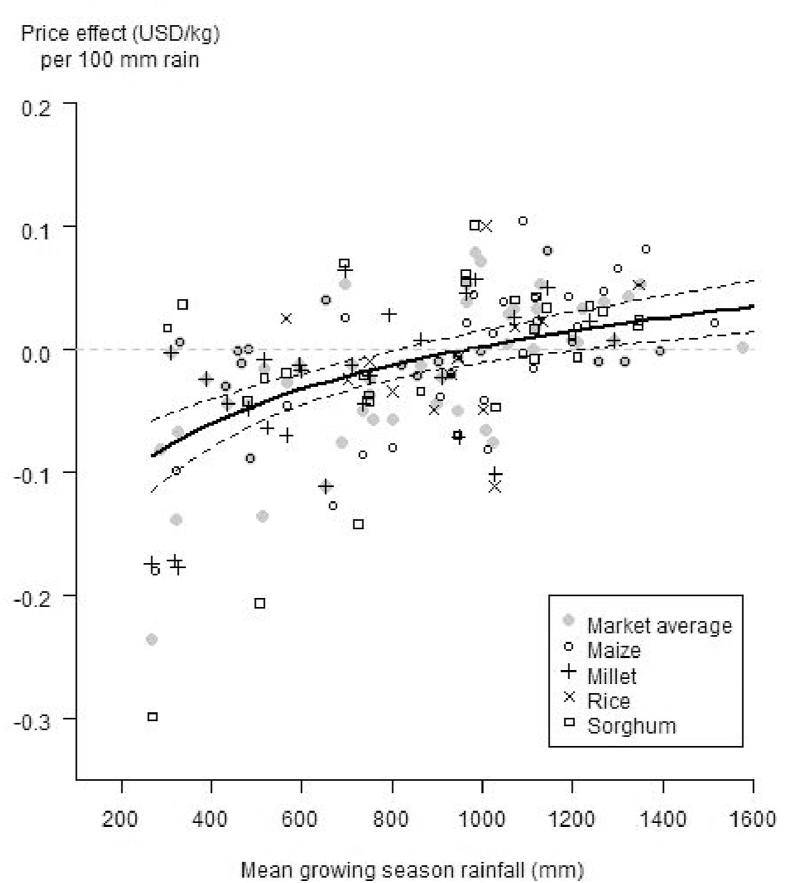
Price effect (USD kg^−1^ per 100 mm of rainfall) per market and crop as a function of average annual precipitation (mm) for 65 markets in sub-Saharan Africa. The regression line shows the estimated price effect (averaged over crops) as a function of the natural logarithm of rainfall. The dashed lines represent the 95% confidence interval, R^2^ = 0.32.

We found a modest effect of precipitation in the preceding growing season (hereinafter “lagged rain”) on food prices. The effect of rainfall was most pronounced when the average growing season rainfall was below 950 mm (Fig. 2). For example, regions with average growing season rainfall of 500 mm, a decrease of 100 mm in rainfall leads to a price increase of USD 0.05. In humid areas, in contrast, we did not find large price effects of lagged rainfall except at very high average rainfall levels, where excessive rainfall may be problematic. The annual price variation was similar for maize, sorghum, and millet, but much less pronounced for rice. It is important to note that rice is not typically grown in very dry areas, and when is it, it is irrigated. We found that the crops had very similar response curves, and we therefore generalized the relationship between cereal prices and rainfall with a single model. In addition, differences between models for different crops cannot directly be interpreted, as not all crops are present in each market. We nevertheless differentiate the observations by crop in Fig. 2 to give a better sense of the underlying data. The markets in areas with less than 350 mm average annual rainfall (Al Fashir and El Obeid in Sudan, Saint Louis in Senegal, Borama in Somalia, Dire Dawa in Ethiopia, and Moussoro in Chad) show a strong price effect, even though rainfed crop production is very limited in these regions. The countries with the most pronounced effect of rainfall on prices (price decreasing with an increase in lagged precipitation) were in the Sahel (Sudan, Mali, and Chad).

### 3.2 Seasonal price variation

**Fig. 3:**
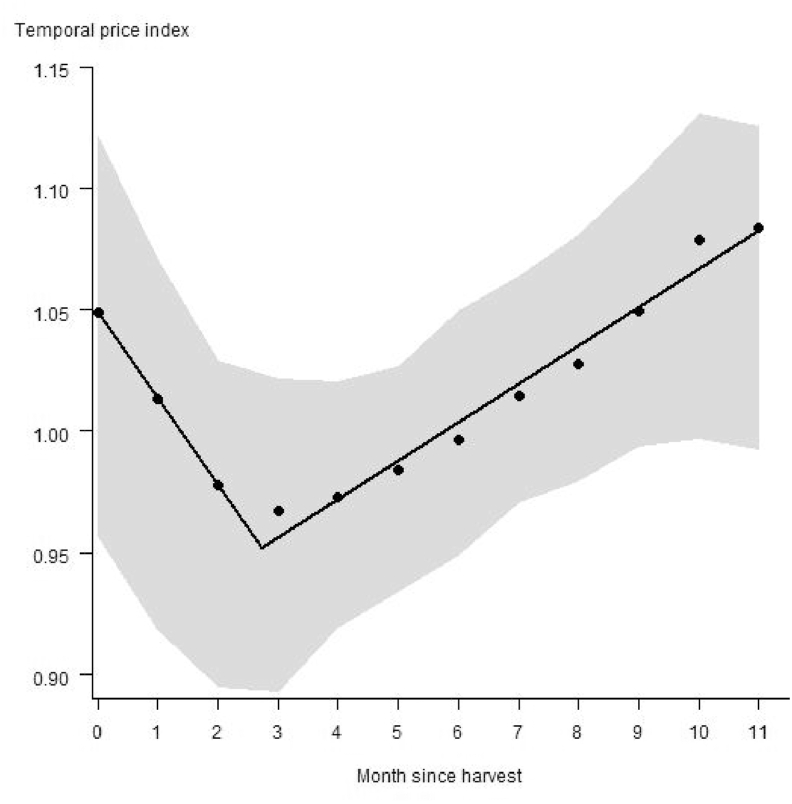
Seasonal food price as a function of month since the beginning of harvest for 160 markets in sub-Saharan Africa. Shown are averages across crops for the four crops considered. The prices are expressed as the median monthly price divided by the annual price. The two regression lines show (1) price decrease after harvest (slope = −0.04) followed by (2) increases until the next harvest (slope = 0.02). The gray envelope represents 70% of the observations.

Food prices in SSA were lowest three months after harvest (96% of the median annual price), and they were highest in the month before harvest (108% of the median annual price). From the lowest price to the month before harvest price increase with a rate of 2% per month (Fig. 3).

The Random Forest model of the temporal price index explained 60% of the variance and the cross-validation correlation coefficient between observed and predicted values was to 0.93. The most important variables in the model were month and rainfall. The temporal RMSE of the model was 0.05 and the RMSE of the null-model assuming a constant price was 0.11. Thus, the model improved accuracy by 56% in comparison with assuming no temporal price variation.

**Fig. 4:**
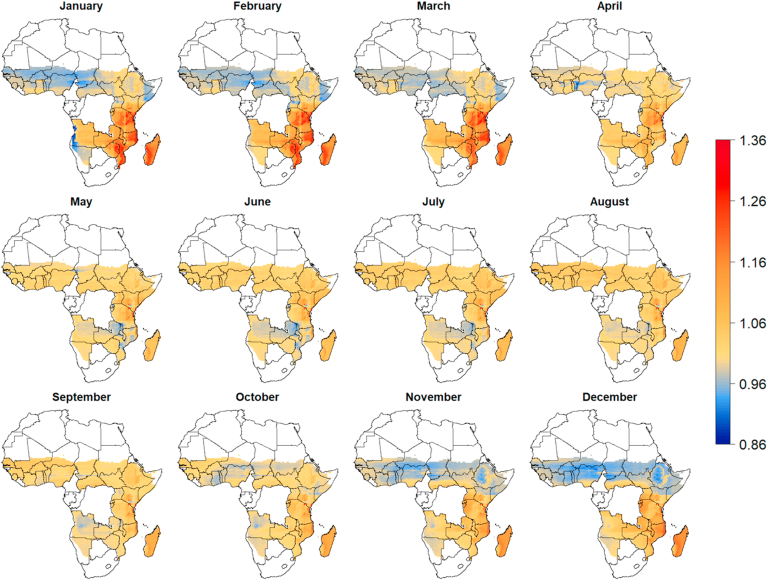
Predicted spatial variation in the seasonal grain prices in Sub-Saharan Africa (SSA). Prices are expressed as the temporal price index is the local median monthly price divided by the annual price. The predictions where made with a Random Forest model using longitude, latitude, month of the year, and annual rainfall as predictor variables. Predictions were made for countries in SSA for which we had price data from local markets, excluding areas with annual precipitation lower than 200 mm.

In the semi-arid regions of west and northeast Africa, harvests are in September and October and prices were low from November to March while they were higher from June to September. In south-east Africa the inverse pattern is visible, with high prices from December to March and lower prices from May to September (Fig. 4).

**Fig. 5:**
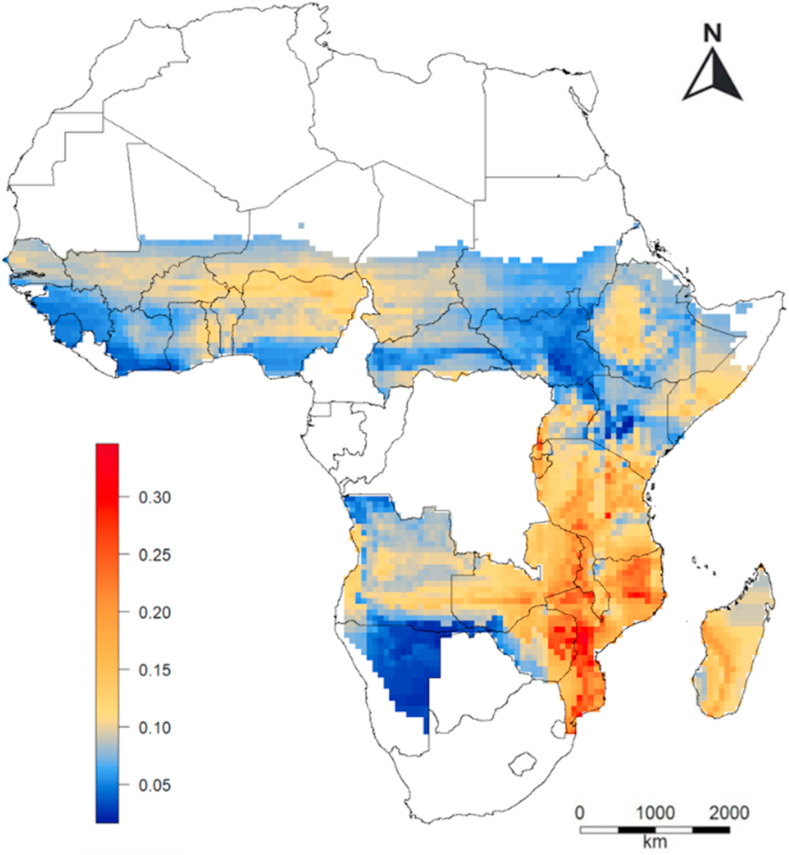
Predicted spatial variation in the annual range of the temporal price index in Sub-Saharan Africa (SSA). The temporal price index was computed by subtracting the median annual price for the mean monthly price. The range represent the difference between the highest and lowest value. Predictions were made for countries in SSA for which we had price data from local markets, excluding areas with annual precipitation lower than 200 mm.

We found strong spatial variation in the magnitude of the seasonal variation (that is, the. difference between the maximum and minimum value of the temporal price index). In South-East Africa it is > 0.25 while it is very low in Kenya and North-Uganda, and in West Africa except north-Nigeria. (Fig. 5).

**Fig. 6:**
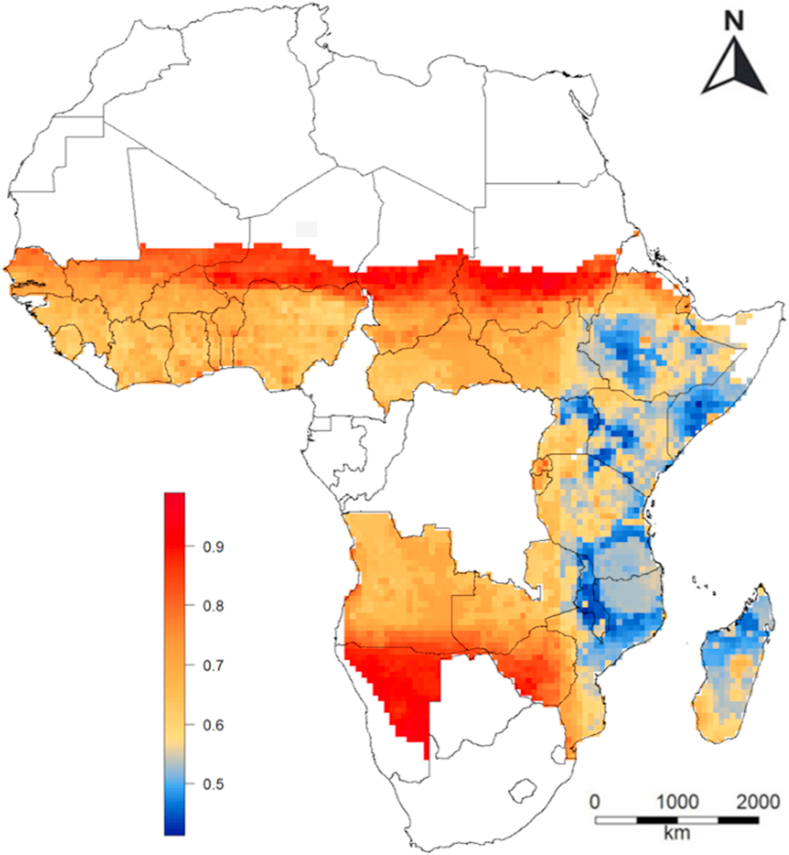
Predicted spatial variation in the spatial maize price (USD kg^−1^) in Sub-Saharan Africa. The predictions where made with a Random Forest model using annual precipitation, and estimated travel time to the nearest town with 20,000 to 50,000 inhabitants, 200,000 to 250,000 inhabitants, and 1 to 5 million inhabitants as predictor variables. Predictions were made for countries in SSA for which we had price data from local markets, excluding areas with annual precipitation lower than 200 mm.

The Random Forest model of spatial price variation explained 42% of the variance, the cross-validation correlation coefficient was 0.68, the RMSE of the model was 0.17 USD kg^−1^ and the RMSE of the null-model assuming a constant price was 0.22 USD kg^−1^. The most important variables in the model were location and rainfall. Prices were higher in West and South-West Africa than East Africa. Prices were highest in very isolated areas (e.g. desserts) and in some areas with very high population density (major cities) in West Africa (Fig. 6).

Annual, seasonal, and spatial price variation.

**Fig. 7:**
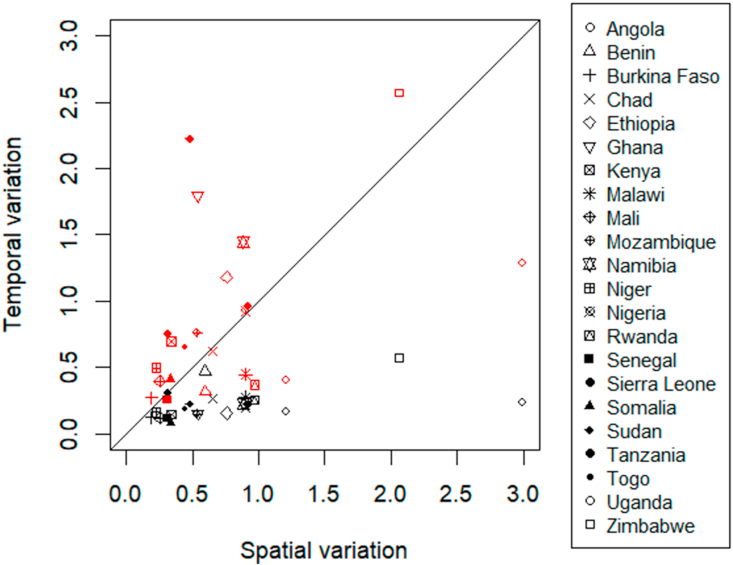
Spatial versus temporal price ranges (USD kg^−1^) by country. Temporal variation for annual (between years; red symbols) and seasonal (within years; black) price ranges. (For interpretation of the references to colour in this figure legend, the reader is referred to the Web version of this article.)

Across countries, average annual price variation was 0.88 ± 0.66 USD kg^−1^, which is larger than the spatial variation (0.76 ± 0.65 USD kg^−1^), and much larger than the seasonal variation (0.22 ± 0.29 USD kg^−1^) (Fig. 7).

## 4 Discussion and conclusions

While there has been a considerable amount of research on food price variation in Africa (e.g. Manda, 2010; Aker, 2008; Gitonga et al., 2013; Hirvonen et al., 2015; Gilbert et al., 2017) there have been no prior efforts at systematic analysis across the continent with a view to generalization that allows estimating prices at unsampled locations and points in time. Our study addresses this gap, using price observations spanning ten years from 168 local markets in 30 countries to describe spatio-temporal patterns in market prices for cereals in SSA. While our modeling results support many of the broad generalizations about price patterns in the region, our detailed description of such patterns at the continental scale is a novel contribution, which suggests good scope for predictive modeling to fill data gaps in local market prices.

At the country level, inter-annual variation was the largest source of price variation in our data, particularly in arid and semi-arid agroecological zones, where local production outcomes are most sensitive to rainfall. In these areas, rainfall in the preceding growing season is associated with price variation for all grains except for rice, which is not grown in very dry areas, and where it is, it is irrigated. Also, much of the rice consumed in Africa is imported from Asia (World Bank, 2012). Further research might also consider the effect of world market prices on national and local prices.

As expected, we found that within-year price variation is strongly related to the cropping cycle, with highest prices in the “lean-season” before harvest, and lowest prices after harvest. We found that the lag between the onset of harvest and maximum market saturation (the lowest price) is two to three months. We also showed that the seasonality is much more pronounced in South-East Africa than in other parts of the continent. The reasons for these geographical patterns are unclear and deserve more study; they possibly reflect the prevalence of short-term maize trade bans in Eastern and Southern Africa (Porteous, 2017).

Understanding food price seasonality in developing countries is important for many reasons. First, seasonality in prices may translate into seasonal variation in dietary intake and nutrition (Dercon and Krishnan, 2000; Kaminski et al., 2014; Kaminski et al., 2014). Secondly, poverty measurement relies heavily on food expenditure information for each household which is typically collected only once a year. Annual expenditures measures derived from these surveys will be incorrect when food price seasonality is substantial and not corrected for (Muller, 2002; Van Campenhout et al., 2015). This should be considered in survey design since a sample which is nationally random may fail to be seasonally random. Third, food buying households face important welfare losses because they are frequently trapped in “sell-low, buy-high” strategies to meet short term cash needs at harvest (Moser et al., 2009;Stephens and Barrett, 2011; Palacios-Lopez et al., 2015; Fink et al., 2018; Burke et al., 2019).

In terms of spatial variability, our results underscore the fact that smallholder farmers in SSA occupy very heterogeneous environments in terms of remoteness from markets, and such remoteness strongly affects farm gate price ratios, price volatility, and other local market characteristics (e.g. Stifel and Minten, 2008; Minten et al., 2013; Chamberlin and Jayne, 2013; Ndiaye et al., 2015; Le Cotty et al., 2017). For example, seasonal variation in food prices on rural African markets is associated with poor infrastructure, and lack of competition in transport and storage (Osborne, 2005; Dillon and Barrett, 2013: Poulton et al., 2006), all of which increase with remoteness (Stifel and Minten, 2008). Moctar et al. (2015) used data on local maize prices in Burkina Faso to show that price volatility increases with remoteness. Investments in rural road infrastructure and in local commercial food storage can reduce price variability (Barret, 1996) and lead to increased consumption and real incomes (Stifel and Randrianarisoa, 2006). Another strand of literature has emphasized trade barriers as a source of increased price volatility (e.g. Porteous, 2017). Our model of spatial price variation explained about half of the spatial variation in maize prices, which suggests that predictive maps of spatial prices are warranted and may help to fill in data gaps related to location-specific market information.

Spatio-temporal price predictions have direct implications for efforts to address food security concerns. Monitoring of food prices, along with production conditions, is a cornerstone of information systems aimed at tracking food security situations, such as the Famine Early Warning Systems Network. Better understanding of how local food prices vary across time and space can support such monitoring efforts as well as the targeting of food security interventions. In addition, predictive price models can enable *ex ante* analysis of the potential food security impacts of policies and public investments in, for example, transportation networks.

10.13039/100014337Furthermore, predictive price models can support policy-oriented microeconomic research. In SSA, facilitating productive investments by smallholder farmers is a considered key to improving agricultural productivity, food security and rural household welfare outcomes. Input and output prices, and their volatility, affect profitability, risk, and the willingness of farmers to invest in agricultural technologies (Foster and Rosenzweig, 2010; Ceballos et al., 2017; Bridle et al., 2020, Michler et al. 2020). Empirical assessments of the incentives driving farmer investments will benefit from more extensive market price data collection. For the time being, price observations will likely remain sparse, and plausible estimates of local prices could be highly valuable. Even with a relatively limited spatial database of prices, our predictive modeling results explain more than half of the observed variation in market prices, suggesting that predicted prices can serve as useful proxies for unobserved local market prices, even where model training data are limited. The “price maps” generated by our predictive models are almost certainly closer to the truth than the assumption that all rural producers face a single spatially-invariant set of prices (often defined for a major urban area, or constructed as an average of available market prices). Given the importance of effective input and output prices in understanding the economic returns of different production strategies pursued by smallholder farmers in different areas, it is important to recognize spatial price variability in analysis.

The degree to which prices can be confidently estimated for a particular region will depend on a number of factors, including the amount, distribution and quality of the observed price data and the amount of price variation, the presence of discernible spatial patterns and the availability of relevant predictor variables. To improve predicted prices in more remote areas, it may be especially important to collect more data from such locations to improve our understanding of transportation cost effects. Given the costs of data collection for large areas such as countries, the targeting of market price collection efforts is important. While the dataset we assembled is large in comparison to prior studies, and has relatively good spatial and temporal coverage, smaller rural markets are under-represented. Collecting price data from smaller markets in remote areas would be an important complement to the price data collected in the larger regional market centers. Such data could help increase our understanding of the influence of isolation on price formation and improve the accuracy of price predictions.

To support such work, there is a need for investments to expand the set of publicly available market price observations across SSA, while assuring that sampling is representative of the region's diverse production and market environments. Taking stock of existing national resources for market information may clarify the scope for national systems to feed into regional systems. The economies of scope and scale in predictive mapping and analysis may incentivize further national-level investments. For example, a country without a current market information system, may be incentivized to establish one if it enabled access to regional prices and improved price predictions within its territory. There would probably still be a need for analysis to be done at the regional level but sharing the costs of that over many contributing countries may make this feasible. Furthermore, given the costs of traditional market information systems, it may be worth exploring alternative data collection measures which may be cost-effective, such as crowdsourcing (Zeug et al., 2017). In addition to national-level efforts, it may be possible to build or extend existing regional systems. Several non-governmental price information systems currently collect data on food prices within the region, e.g. FAO's Global Information and Early Warning System (GIEWS), FEWS NET, the Eastern African Grain Council's Regional Agricultural Trade Intelligence Network (RATIN). Such systems could be extended to include predictive mapping. One caveat of our study is that we observe market prices, not farmgate prices. As such, our spatial price prediction is the expected market price for a given location if there were a market in that location. Given non-trivial “last-mile” transportation and other transfer costs between local markets and farm locations, the effective prices that farmers face at their production locations may differ considerably from prices at the nearest market (e.g. Minten et al., 2013). In the case of prices for marketed grain output, this would be the market price net of the transfer costs; in the case of grain for household consumption, this would be the market price plus the transfer costs. Additional data collection and modeling may usefully be directed at estimating farmgate prices from observed (or estimated) local market prices.

## Declaration of competing interest

The authors declare no conflicts of interest.
