# Supplementary material for: Seasonal, annual, and spatial variation in cereal prices in Sub-Saharan Africa
Source: Glob Food Sec. 2020 Sep;26:100438. doi: 10.1016/j.gfs.2020.100438 (PMC7727398; doi:10.1016/j.gfs.2020.100438)
Supplement: Multimedia component 1 [file mmc1.docx]

# Appendices

## Appendix A: Description of price data

**Table 1S:** Overview of the compiled cereal price data (43,399 records) by country. Number of markets (n); mean price; standard deviation (sd) price (USD kg^-1^).

|  | **Maize** | | | **Millet** | | | **Sorghum** | | | **Rice** | | |
| --- | --- | --- | --- | --- | --- | --- | --- | --- | --- | --- | --- | --- |
| **Country** | **n** | **mean** | **sd** | **n** | **mean** | **sd** | **n** | **mean** | **sd** | **n** | **mean** | **sd** |
| Angola | 3 | 0.56 | 0.13 | 0 | -- | -- | 0 | -- | -- | 1 | 3.81 | 1 |
| Benin | 4 | 0.82 | 0.19 | 0 | -- | -- | 4 | 1.30 | 0.44 | 0 | -- | -- |
| Burkina Faso | 0 | -- | -- | 8 | 0.85 | 0.14 | 8 | 0.74 | 0.11 | 0 | -- | -- |
| Burundi | 1 | 0.92 | 0.24 | 0 | -- | -- | 0 | -- | -- | 0 | -- | -- |
| Central African Republic | 1 | 0.97 | 0.39 | 0 | -- | -- | 0 | -- | -- | 0 | -- | -- |
| Chad | 11 | 0.95 | 0.25 | 11 | 1 | 0.25 | 12 | 0.74 | 0.22 | 11 | 1.95 | 0.51 |
| Cote d’Ivoire | 0 | -- | -- | 0 | -- | -- | 0 | -- | -- | 1 | 1.85 | 0.08 |
| Djibouti | 0 | -- | -- | 0 | -- | -- | 0 | -- | -- | 1 | 1.1 | 0.3 |
| Ethiopia | 6 | 0.88 | 0.64 | 0 | -- | -- | 3 | 1.15 | 0.75 | 0 | -- | -- |
| Ghana | 5 | 0.76 | 0.43 | 5 | 1.24 | 0.68 | 5 | 0.95 | 0.54 | 4 | 1.9 | 0.99 |
| Guinea | 0 | -- | -- | 0 | -- | -- | 0 | -- | -- | 1 | 1.59 | 0.35 |
| Kenya | 6 | 0.67 | 0.27 | 0 | -- | -- | 0 | -- | -- | 0 | -- | -- |
| Madagascar | 0 | -- | -- | 0 | -- | -- | 0 | -- | -- | 1 | 1.99 | 0.74 |
| Malawi | 13 | 0.46 | 0.21 | 0 | -- | -- | 0 | -- | -- | 12 | 1.59 | 0.61 |
| Mali | 0 | -- | -- | 6 | 0.86 | 0.17 | 6 | 0.78 | 0.15 | 6 | 1.7 | 0.27 |
| Mozambique | 9 | 0.52 | 0.23 | 0 | -- | -- | 0 | -- | -- | 8 | 1.36 | 0.36 |
| Namibia | 0 | -- | -- | 3 | 1.52 | 0.52 | 4 | 2.56 | 1.12 | 8 | 2.69 | 0.7 |
| Niger | 4 | 1.04 | 0.20 | 6 | 0.94 | 0.16 | 6 | 0.87 | 0.14 | 0 | -- | -- |
| Nigeria | 16 | 0.64 | 0.29 | 16 | 0.69 | 0.29 | 16 | 0.68 | 0.31 | 16 | 1.51 | 0.82 |
| Rwanda | 4 | 0.97 | 0.50 | 0 | -- | -- | 0 | -- | -- | 1 | 2.34 | 0.49 |
| Senegal | 0 | -- | -- | 11 | 0.9 | 0.14 | 2 | 0.99 | 0.13 | 0 | -- | -- |
| Sierra Leone | 0 | -- | -- | 0 | -- | -- | 0 | -- | -- | 4 | 1.53 | 0.34 |
| Somalia | 4 | 0.28 | 0.19 | 0 | -- | -- | 0 | -- | -- | 0 | -- | -- |
| South Sudan | 1 | 0.53 | 0.50 | 0 | -- | -- | 0 | -- | -- | 0 | -- | -- |
| Sudan | 0 | -- | -- | 8 | 1.36 | 0.79 | 8 | 0.97 | 0.64 | 0 | -- | -- |
| Tanzania | 4 | 0.69 | 0.24 | 0 | -- | -- | 0 | -- | -- | 1 | 2.44 | 0.6 |
| Togo | 6 | 0.71 | 0.23 | 0 | -- | -- | 5 | 0.88 | 0.36 | 0 | -- | -- |
| Uganda | 7 | 0.59 | 0.27 | 0 | -- | -- | 0 | -- | -- | 1 | 2.34 | 0.58 |
| Zambia | 1 | 0.68 | 0.34 | 0 | -- | -- | 0 | -- | -- | 0 | -- | -- |
| Zimbabwe | 2 | 0.66 | 0.45 | 0 | -- | -- | 0 | -- | -- | 10 | 2.57 | 0.93 |
| **Total/mean** | **108** | **0.72** | **0.31** | **9** | **1.04** | **0.35** | **12** | **1.05** | **0.41** | **17** | **2.02** | **0.57** |

## Appendix B: Cereal price relationship

We fitted linear regression models to study the relationship between maize price and the other cereals prices (millet, rice, and sorghum). For maize and millet (maize price=0.82*millet price), the RMSE was 0.22 USD kg^-1^ and the correlation coefficient was 0.61. For maize and sorghum (maize price = 0.78*sorghum price), the RMSE was equal to 0.25 USD kg^-1^ and the correlation coefficient was 0.69. For maize and rice (maize price= 0.37*rice price), the RMSE was 0.22 USD kg^-1^ and the correlation between observed maize price and predicted was 0.58 (Figure 1S).


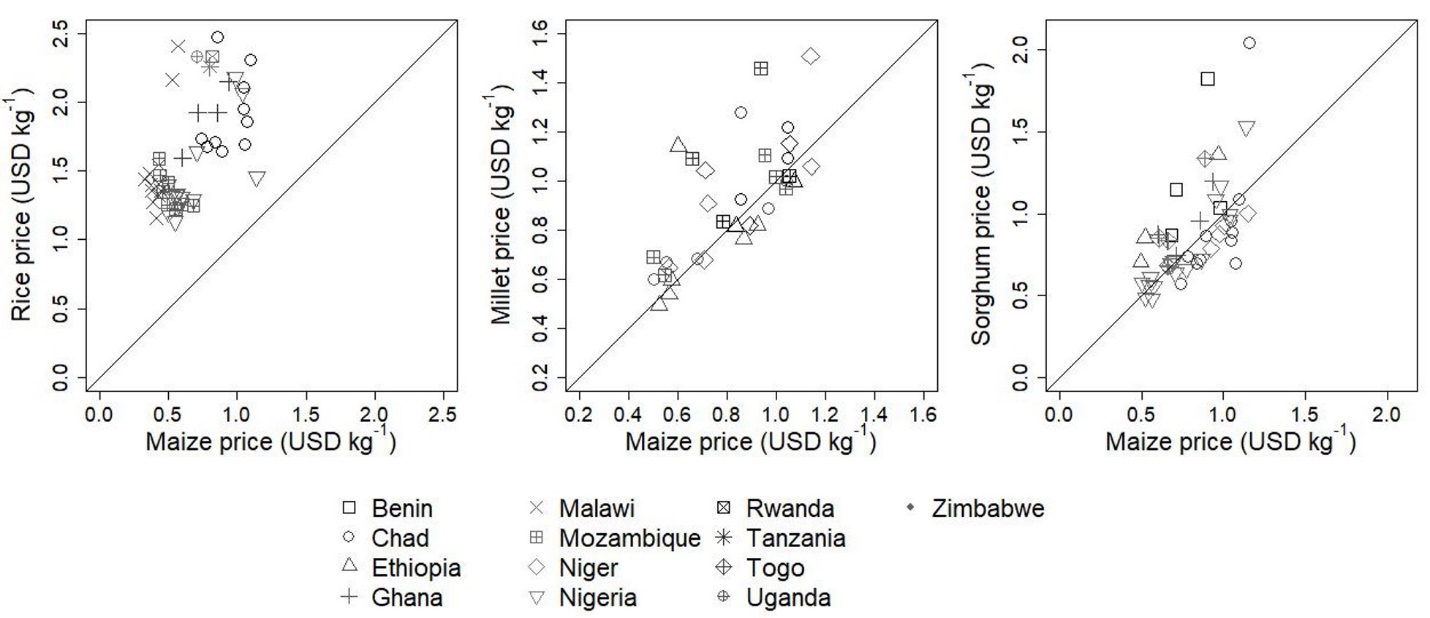


**Figure 1S:** Average maize price (USD kg^-1^) versus average millet price, rice price, sorghum price (USD kg^-1^) for markets where both prices were reported.

## Appendix C: Determination of the end of the growing season

endSeason <- function(x, th=30){

cumfun <- function(x) {

test <- x == 1

y <- cumsum(test)

y - cummax(y * !test)

}

if (any(is.na(x))) return (rep(0, 12))

med <- median(x)

th <- min(90, max(th, med))

xth <- c(x[7:12], x, x[1:6]) >= th

z <- cumfun(xth)[7:18]

y <- z > 5

if (sum(y) == 0) {

y <- z > 4

}

if (sum(y) == 0) {

y <- z > 3

}

if (sum(y) == 0) {

y <- z > 2

}

y

}
